# Anecdote of Thomas Paine

**Published:** 1863-04

**Authors:** Grant Thorburn

**Affiliations:** New Haven


### ANECDOTE OF THOMAS PAINE.

In 1794, when Paine and Robespierre were establishing Liberty in France by means of the guillotine, the strife between them was, who shall be the greatest—Robespierre got the ascendant, and Paine, with twenty others, were enrolled for the guillotine. On the basement, was a row of cells running the whole length of the prison; one prisoner was confined in each cell. Every night, the Clerk of the Tribunal came round with a list, and marked a cross with chalk on the door of the person who was to be guillotined in the morning. That night, the clerk had a list containing twenty-one names, with Paine's among them. The keeper of the prison stood in Paine's door conversing; thus Paine's door stood open against the wall *inside out.* The clerk (probably being drunk) not observing, marked Paine's door; in the morning, the executioner, having emptied the cells whose doors were marked (Paine being in bed, and the door shut, no mark appeared), he fell one short. "Never mind," said he to the guards, " we'll take the next!" In forty-eight hours thereafter, Robespierre's head rolled in the basket, and thus Paine escaped. * * * Says I, " Mr. Paine, I would call this a providential deliverance; what did you think?" Said he, "I thought the *Fates* had ordained I was not to die at that time?" Says I, "Neither you nor any of the wise men in the East, can tell what *Fates* mean; you wrote much against the religion of the Bible; you highly extolled the perfectibility of man, when left untrammeled by priestcraft and dogmas. I think God spared your life, that you might show to the world, and to your friends in America in particular, what man is, when left by his Maker to wander in his own counsels. Here you sit, in a dirty, uncomfortable room, bedaubed with snuff, and stupefied with brandy—you, who were once the companion of Washington, Jay, and Hamilton, are now shunned by every respectable man; and I have seen superfine-coat infidels cross the street, to avoid your recognition." Says he, "I care not a straw what the world says about me." "Then," says I, "I envy not your feelings; I wish so to conduct myself, that I may gain the good will of my fellow men." GRANT THORBURN, SEN.

New Haven, 27th July, 1859.

